# Improving hand-eye-coordination in laparoscopy using a virtual endoscope monitor

**DOI:** 10.1007/s11548-026-03620-4

**Published:** 2026-04-25

**Authors:** Bernhard Michael Weber, Mathilde Connan, Alexander Kirst, Dominik Schneider, Luca Wegener, Maximilian Berlet, Jonas Fuchtmann, Dirk Wilhelm, Julian Klodmann

**Affiliations:** 1 Institute of Robotics and Mechatronics, German Aerospace Center, Muenchener Str. 20, 82234 Wessling, Germany; 2 Research Group Minimally Invasive Interdisciplinary Therapeutical Interventions (MITI), Technical University of Munich, Trogerstr. 10, 81675 Munich, Germany; 3 TUM School of Medicine and Health, Technical University of Munich, Klinikum Rechts der Isar, Department of Surgery, Ismaninger Str. 22, 81675 Munich, Germany

**Keywords:** Laparoscopy, Hand-eye-coordination, Augmented reality, Head-mounted display, User study

## Abstract

**Purpose:**

While laparoscopic procedures offer considerable advantages for the patient, they pose substantial challenges for the surgeon, whose hand-eye coordination is impaired. One reason for this is the surgeon’s lost visual access to the surgical field, who is instead provided with an endoscopic image on a stationary monitor. In the present study, an optical see-through head-mounted display (OST-HMD) was used to display a virtual endoscope monitor. Unlike stationary monitors, this allows the endoscope image to be projected at arbitrary positions in the room.

**Methods:**

The benefits of this approach were evaluated in a user study. *N* = 10 surgeons carried out a threading task in a pelvic trainer integrated in an operating room setup. Four positions of the endoscope image were compared: frontal view ($0^{\circ }$), offset by $60^{\circ }$ to the left and right, and projected directly onto the surgical field.

**Results:**

The results show that for complex bimanual tasks, projecting the endoscope image onto the surgical field led to a halving of execution times in the study. In addition, the forces applied during threading were measured, but no significant effects of perspective were found. More experienced surgeons also reported significantly lower mental workload with the frontal view ($0^{\circ }$) and the projection onto the surgical field compared to the other conditions.

**Conclusion:**

This study provides first evidence that an OST-HMD could be a suitable way for flexibly displaying endoscopic images in laparoscopy and could help improve the surgeon’s hand-eye coordination, while at the same time being easily integrated into the clinical environment.

## Introduction

Laparoscopy has a history dating back over a century and developed from a purely diagnostic procedure, particularly in the second half of the 19th century, into a surgical method [1]. In the last decades, laparoscopy has enjoyed considerable success and widespread use. Today, operations like colectomies, cholecystectomies or proctectomies are routinely performed laparoscopically, although robot-assisted surgery is becoming increasingly relevant [2]. This success is also because the method truly embodies the medical ethical principle of “primum non nocere, secundum cavere, tertium sanare” (first, do not harm, second, be cautious, third heal). The advantages are obvious: the minimally invasive access to the surgical field causes less harm to the patient’s body and significantly shortens the postoperative recovery period.

However, these advantages come at a cost, as the procedure is significantly more demanding for the surgeon compared to open surgery. Direct manual and visual access to the surgical site is not given due to the small abdominal incisions and the use of laparoscopic instruments. Hence, hand-eye coordination is substantially impaired. While the surgeon directly observes the operative field and his/her hands during open surgery, an endoscopic picture of the surgical field and the instruments are displayed on a monitor during laparoscopy. This leads to a “mislocation” of visual information [3]. Additionally, the line-of-sight of the endoscope and the surgeon’s line-of-sight during open surgery usually are divergent, which has been labeled as “misorientation” [3].

Particularly given that manual guidance of laparoscopic instruments through the incision points leads to further disturbing motion effects (mirroring and scaling) and the fact that haptic perception is limited [4], it is essential to minimize the visual effects as much as possible. Empirical studies show that the overall configuration of the surgeon’s position at the table, placement of trocars, endoscope, and monitor have a significant impact on surgeon’s mental and physical workload as well as on surgical performance (e.g., [5–8]). In clinical practice, these detrimental effects are often reduced by selecting appropriate optics, positioning the endoscope between the surgeon’s hands, and positioning the monitor directly opposite the surgeon. However, this approach often restricts the surgeon’s working space or cannot be implemented depending on the surgical procedure and the operation room layout.

A few studies have addressed the question of how the relative position of the monitor affects performance in basic surgical tasks. It was found that positioning the monitor directly opposite to the surgeon (frontal view, $0^{\circ }$) is preferable, and that a lateral offset to the left or right (e.g.,±6$0^{\circ }$ [7]) leads to increased execution times and decreased task performance during threading [9], pick-and-place tasks [7] or knotting [8]. Additionally, studies showed that surgical performance can be further improved by positioning a frontal view monitor on hand-level compared to the conventional eye-level [9, 10] as it further reduces the mislocation effect. In these studies, the hand-level monitor was positioned directly behind the laparoscopic setups, which is difficult to implement in a real surgical setup.

A promising alternative to stationary monitors is the use of head-mounted displays (HMDs), which allow for much greater flexibility. HMDs eliminate the need to look at a monitor, which may be located at an inconvenient position [11]. The endoscopic image can be displayed and adjusted in any position using an HMD. Optical see-through head-mounted displays (OST-HMDs; e.g., Microsoft HoloLens™) are particularly promising in this regard, as they allow the real environment to remain visible while also displaying virtual content (such as the endoscopic image). Another major advantage is the stereoscopic display which allows depth information to be conveyed in the endoscopic image.

In a recent study, the approach of using an OST-HMD for displaying a virtual endoscopic monitor was successfully implemented by the authors and its benefits were validated in a pilot study [12]. Nineteen surgically inexperienced participants performed simple line tracking tasks with the HMD displaying the virtual monitor in two different positions (divergent vs. matched line-of sight of subject and endoscope). The results provided first evidence that an optimal positioning of the virtual endoscope image significantly improves hand-eye coordination and consequently led to better task performance, lower mental load and improved usability. Although these results are encouraging, it remains unclear to what extent these benefits can be translated to the clinical context and trained surgeons, who learned to perform laparoscopic procedures safely and effectively despite impaired hand-eye coordination.

In the present study, the technical approach of the virtual endoscope image, displayed in an OST-HMD, was set up in an operating room, and a clinically more relevant task (inserting a surgical needle through target eyelets) was performed laparoscopically by surgeons. Task performance and workload were compared for four different virtual endoscope positions. In consultation with clinical experts and in line with previous research (e.g., [7]), a frontal view ($0^{\circ }$, eye-level) was compared with laterally offset positions ($\pm \,$6$0^{\circ }$, eye-level). In addition, direct projection onto the surgical field was implemented ($0^{\circ }$, torso-level), which is only possible with an AR approach and eliminates the mislocation effect described above. It was hypothesized that this projection onto the operational field would yield highest task performance and lowest workload, followed by the frontal position and that the lateral positions would lead to the worst results.

Hence, the main contributions of the present work are: (1) Contrary to the cited pilot study with surgical non-professionals [12], the approach of using an OST-HMD for displaying the endoscopic image is tested in a clinical context with realistic tasks performed by surgeons. (2) As an extension of the cited studies investigating the effects of monitor positions [7–10, 12], the current study is the first investigating whether a direct projection of the endoscopic image onto the surgical field improves task performance compared to eye-level positions.

## Methods

### Sample

Ten surgeons (4 female, 6 male subjects; all right-handed) with an average age of M = 40.6 years (SD= 13.4 yrs.; age ranging from 27 to 70 yrs.) participated in the present study. The surgeons were all specialized in the field of visceral surgery. All participants had surgical experience, although a large range of laparoscopic operations were reported. Three of the ten participants can be labeled as novices (5–10 procedures), while the others were more experienced (50–10.000 procedures). Participation in the study was unpaid and voluntary.

### Apparatus

#### Experimental setup

**Fig. 1:**
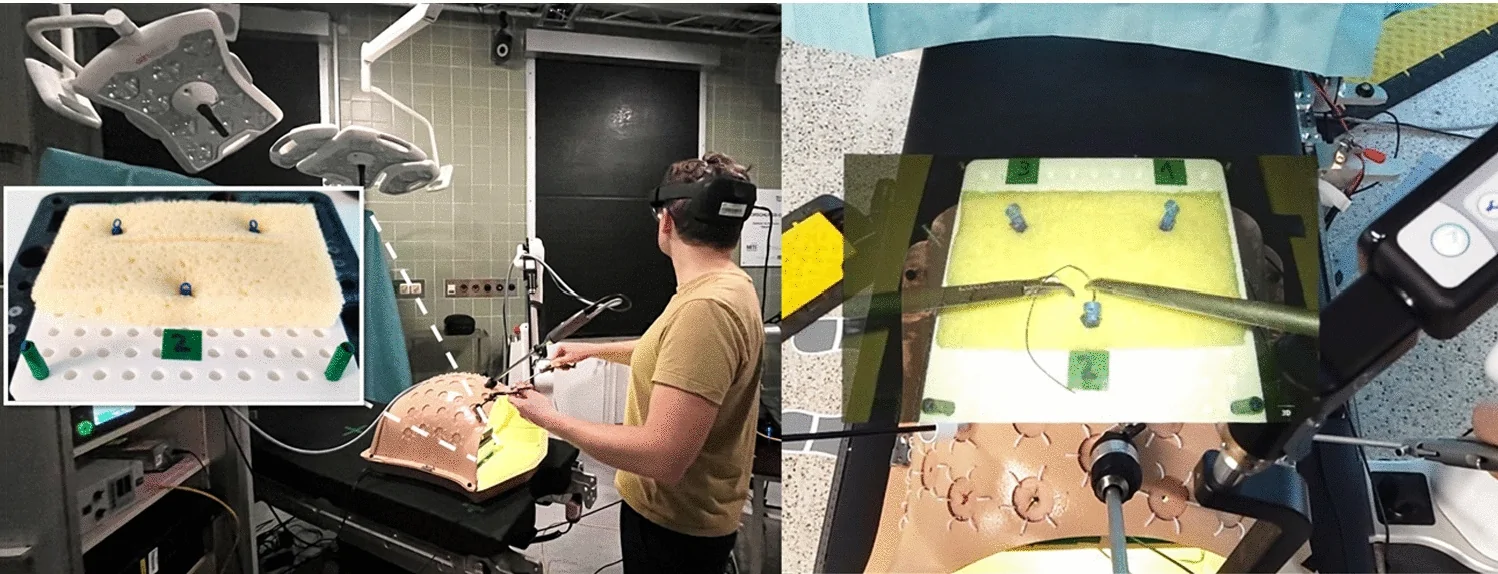
Experimental Setup. Left: Surgeon performing the experimental task in the +6$0^{\circ }$ virtual monitor position. Zoom-out window: Experimental task board with the three blue eyelets and the two green starting position pins. Right: HMD view in Torso projection condition

The study was conducted in an experimental operating room at the TUM Rechts der Isar Hospital. A phantom of an insufflated abdomen (LapTrainer^1^) was positioned on an operating table in this OR. This LapTrainer had various openings for the placement of trocars. A test pad with three blue-colored eyelets in different orientations ($0^{\circ }$,±45$^{\circ }$ relative to the endoscope view; see Fig. 1, left panel) was positioned inside the LapTrainer. The three eyelets were labeled from right to left with the numbers 1, 2 and 3. To avoid hard contacts between laparoscopic instruments and the surface of the test pad, it was coated with a soft, sponge-like material. In addition, two green pins were attached to the left and right sides of the test pad facing the surgeon, which acted as starting points for the instrument tips. The test pad was mounted on a Force-Torque sensor (ATI Nano 43^2^), which was firmly attached to the base of the LapTrainer. In the right hand, a needle holder (KARL STORZ Makro-Needleholder, 5 mm, ${33}{\hbox {cm}}$^3^) and in the left hand, a grasping forceps (KARL STORZ CLICKline grasping forceps$^3$) were used to perform the experimental tasks. A surgical needle (COVIDIEN™  Surgilon™  C-13^4^), with an approx. 5cm long thread was used for the experiment. A stereoscopic endoscope KARL STORZ TIPCAM^®^1 Rubina^®^ 4K/3D$^3$ was used for the study, which was held by an SOLOASSIST^II^^5^ robotic camera control system, to have a standardized endoscope position. The endoscope was oriented in conjunction with the instruments to match the perspective in a laparoscopic setting. Furthermore, the view was adjusted so that the test pad was well covered. The endoscopic video stream was displayed in a Microsoft HoloLens2™ 6 HMD (FoV: 43$^{\circ }$ horizontal, 29$^{\circ }$ vertical; resolution: 1440x936 per eye; integrated eye tracking to optimize 3D visualization, power supply via cable). The HoloLens2 HMD is a pair of mixed reality glasses that weigh 566g. The see-through visor allows an unobstructed view of the real environment, while virtual objects can be displayed in 3D at the same time. The endoscopic video stream as well as the data from the force-torque sensor were recorded during the experimental trials.

#### Experimental software

**Fig. 2:**
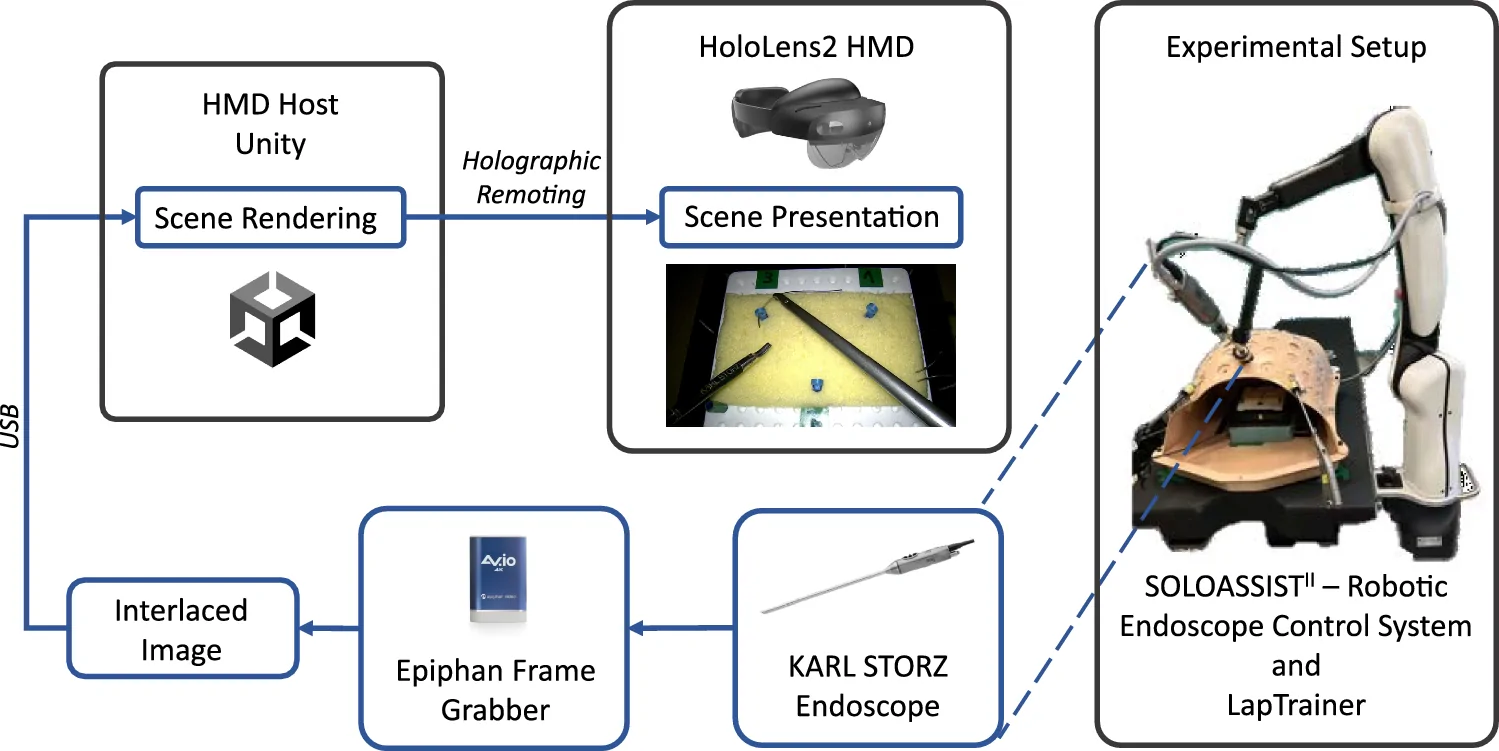
The system architecture with the transfer of endoscopic images to the HMD

The HMD host machine ran Unity and managed the processing of the scene as shown in Fig. 2. Via an Epiphan^7^ AV.io 4K™  frame grabber, the endoscope provided 4K stereo interlaced images, which were split into two distinct HD (1920x1080) channels by the HMD host machine. A fragment shader, implemented on GPU, determined the corresponding image to be rendered on the left and right HMD display after correcting the off-center perspective to define the rendering target for stereo vision. It utilized Holographic Remoting,^8^ to forward the rendered scene to the HMD. Subsequent testing demonstrated that the setup maintains a stable update rate of over 60Hz, fully utilizing the maximum display capabilities of the HoloLens (cf. [12] for further details).

### Experimental task and design

One surgical task that is relevant for a wide range of interventions is laparoscopic suturing. In the present study, this task was abstracted to the insertion of a surgical needle through target positions in order to ensure a high degree of experimental standardization and replicability. Specifically, surgeons had to pass the surgical needle through the three eyelets (1-2-3) as quickly as possible, but at the same time with minimal contact force. The needle should always be inserted in such a way that the tip of the needle pointed toward the surgeon. Therefore, the last eyelet 3 was the most demanding task. Due to its orientation and position the surgeons had to re-grip and re-align the needle several times to successfully complete the task. There were four experimental perspective conditions with different virtual monitor positions relative to the surgeon: (1) Front view ("$0^{\circ }$"), (2) shifted by 60 degrees to the left ("$-\,60^{\circ }$") and (3) to the right ("$+\,60^{\circ }$"), all three on eye-level. Finally, (4) projection onto a horizontal plane at the height of the LapTrainer ("Torso", see Fig. 1 right panel), generating the impression of looking directly onto the test pad. The order of these four experimental conditions was counterbalanced across subjects to control for potential order effects. Yet, the order of the three eyelet trials was fixed (1-2-3).

### Procedure

In a first step, the test subjects were informed about the background of the study, the experimental task and the experimental procedure in a briefing. Afterward they provided written informed consent. In a demographic questionnaire they indicated their age, gender, field of surgical expertise, number of laparoscopic operations and handedness. Then the surgeons adjusted the height of the operating table so that it was comfortable for them. They reported their body height and the virtual endoscope monitor position was accordingly set in a way that the upper edge of the video stream matched the subject’s eye level for the $0^{\circ }$ and $\pm \,60^{\circ }$ conditions. The HMD was put on and the straps of the HMD were tightened so it was safe and comfortable to wear. In addition, the interpupillary distance was individually adjusted to ensure optimal 3D vision. The ceiling lighting was then dimmed to a predefined brightness value for optimal visibility of the virtual endoscope image in the HMD. The subjects were then familiarized with the experimental task in a warm-up trial. For this trial, the $0^{\circ }$ perspective was chosen. The tips of the two forceps were positioned at the defined starting points (green pins) and when they indicated to be ready, the trial was started. The three eyelet tasks were performed one after the other. Subsequently, subjects reported their mental and physical workload. The corresponding items of the Surgery Task Load Index questionnaire (SURG-TLX) [13] were used for this purpose (1. “Mental demands: How mentally fatiguing was the procedure” and 2. “Physical demands: How physically fatiguing was the procedure”, both 20-point scales ranging from 1 = “very low” to 20 = “very high”). Then, the main experiment was started, following the very same procedure. For each perspective the three eyelets were completed two times, while the first time was considered as a training trial. Thus, a total of 4 (perspectives) × 3 (Eyelets) × 2 (Repetitions) = 24 experimental trials were completed. The workload ratings were always given after completion of a perspective condition, i.e., after having completed the three eyelets two times. After the experiment, surgeons briefly reported their overall impression regarding the use of an OST-HMD for displaying the endoscopic video in laparoscopy.

### Data analysis

During data analysis, endoscopic video recordings and force data from the Force-Torque sensor (sampling rate: 100Hz) were used. The video data were segmented to identify the time intervals during which the needle interacted with each eyelet, with an interaction considered complete once the needle (excluding the suture thread) had fully passed through the eyelet. Segmentation was performed using Shotcut,^9^ and temporal markers were exported for each configuration ($0^{\circ }$, $+\,60^{\circ }$, $-\,60^{\circ }$, Torso), repetition (first and second trial), and eyelet (1,2,3), including the corresponding start and end times of each interaction. Force data were calibrated by subtracting the initial sensor value from all subsequent measurements. Additionally, only force values exceeding a threshold of 0.03 N were considered, ensuring that periods in which the needle was not interacting with the eyelet were excluded. For each eyelet interaction the root mean square error (RMSE) of forces was calculated.

For all measures, normality was checked using the Shapiro-Wilk test. In case of normality, a repeated measures 4 (perspective) x 3 (eyelet) ANOVA (rmANOVA) was performed. For the SURG-TLX ratings a rmANOVA was performed on the mental and physical demand ratings in the four perspective conditions. Sphericity was tested using Mauchly’s test, and Greenhouse-Geisser correction were made if sphericity was not given. Alpha levels of post-hoc comparisons were Bonferroni corrected. Additionally, Hedges’ g was calculated as effect size measure.

## Results

**Table 1:** Means (SD in parentheses) for durations, interaction forces and workload ratings

Perspective	${-\,60}^{\circ }$	${0}^{\circ }$	+ 60$^{\circ }$	Torso
*Durations [sec]*				
Eyelet 1	17.6 (7.9)	15.0 (3.6)	23.2 (11.1)	17.6 (6.7)
Eyelet 2	15.3 (2.6)	17.5 (8.5)	23.2 (13.8)	15.6 (6.4)
Eyelet 3	31.9 (24.1)	26.5 (16.9)	35.7 (25.7)	15.5 (4.0)
*Force RMSE [N]*				
Eyelet 1	1.29 (0.49)	1.09 (0.42)	1.26 (0.42)	1.32 (0.52)
Eyelet 2	1.00 (0.29)	0.91 (0.36)	0.97 (0.35)	1.20 (0.46)
Eyelet 3	1.12 (0.35)	1.30 (0.52)	1.07 (0.38)	1.08 (0.40)
*Workload [1-20]*				
Mental	9.8 (4.3)	7.0 (4.4)	8.6 (2.7)	7.0 (3.1)
Physical	6.6 (3.4)	4.9 (4.0)	7.6 (3.9)	6.8 (4.9)

### Durations

In a first step of analysis, an outlier analysis was performed and revealed two participants with extreme outlier values (with durations >100 sec for single eyelets). These cases represented less experienced surgeons who had severe problems completing the task, independently from the perspective condition. Next, normality was tested and non-normality was indicated by Shapiro-Wilk tests (W < 0.71). Hence, nonparametric analyses were performed. A first Friedman test was performed to check whether there are overall effects of the perspective on durations. Yet this was not the case for any of the three eyelets (all $\chi ^2$s(3) < 4.7). Directly comparing the differences between ± $60^{\circ }$ and $0^{\circ }$, however revealed that for eyelet 1 there was a significant difference between $+\,60^{\circ }$ (*M* = 23.2 sec; SD = 11.1 sec, Table 1) and $0^{\circ }$ (*M* = 15.0 sec; SD = 3.6 sec, Wilcoxon’s Z = 2.5; *p* = 0.024, one-tailed testing; Hedges’ g = 0.99). Moreover, for eyelet 3, there was a trend for shorter durations in the "Torso" condition (*M* = 15.5 sec; SD = 4 sec) compared to the $+\,60^{\circ }$ condition (*M* = 35.7 sec; SD = 25.7 sec; *Z* = 2.0; *p* = 0.10, one-tailed testing; Hedges’ g = 1.10) and the $-\,60^{\circ }$ condition (*M* = 31.9 sec; SD = 24.1 sec; *Z* = 2.1; *p* = 0.07, one-tailed testing; Hedges’ g = 0.95). Although these comparisons did not reach the conventional level of significance the effect sizes were large.

### Interaction forces

Mean force data were normally distributed (Shapiro-Wilk’s W >.84). For these following analyses, the two outlier cases were again omitted. The rmANOVA on the force data, however, revealed no significant main effects (Perspective: F (3, 21) = 0.88; Eyelet: F (2, 14) = 2.04, ns.) or interaction effect (F (6, 42) = 0.90, ns.).

### Workload

*Mental workload*: First, the mental demands ratings of the SURG-TLX questionnaire were analyzed. Normality was checked with the Shapiro-Wilk test, which revealed no significant deviations from normality (all Ws >0.88). The rmANOVA on these ratings revealed a significant overall effect of perspective (F (1.65; 14.83) = 4.63; *p* = 0.03, $\eta ^2$ = 0.34). Overall, the ratings were higher for the $-\,60^{\circ }$ (*M* = 9.8; SD = 4.3) and $+\,60^{\circ }$ (*M* = 8.6; SD = 2.7) perspectives, compared to the $0^{\circ }$ (*M* = 7; SD = 4.4) and torso (*M* = 7; SD = 3.1) perspectives, although these differences did not reach significance in post-hoc comparisons. Excluding the three novices from this analysis, however, revealed a larger overall effect (F (3; 18) = 9.19; *p* = 0.001, $\eta ^2$ = 0.61) and significant post-hoc comparisons. Specifically, the ratings for $0^{\circ }$ (*M* = 5.9; SD = 3.1) was significantly lower compared to the $-\,60^{\circ }$ condition (*M* = 10.0; SD = 4.9; *p* = 0.04; one-tailed testing; Hedges’ g = 1.0); comparing the $0^{\circ }$ and the $60^{\circ }$ conditions (*M* = 8.5; SD = 2.8), however, did not reveal a significant difference (*p* = 0.07, one-tailed testing; Hedges’ g = 1.0). Similarly, the mental demand ratings during the Torso condition (*M* = 6.1; SD = 2.6) were significantly lower compared to the $-\,60^{\circ }$ condition (*p* = 0.03, one-tailed testing; Hedges’ g = 0.99) and tended to be lower compared to the $60^{\circ }$ condition (*p* = 0.05, one-tailed testing; Hedges’ g = 0.89).

*Physical workload*: As normality was not given for the ratings (Shapiro-Wilk’s W <.79), nonparametric analyses were conducted in this case. Friedman test actually yielded a significant overall effect of perspectives ($\chi ^2$(3) = 9.4; *p* = 0.02). Post-hoc Wilcoxon tests comparing the four perspectives showed that the physical demand ratings were significantly higher in the $+\,60^{\circ }$ condition (*M* = 7.6; SD = 3.9) compared to the $0^{\circ }$ condition (*M* = 4.9; SD = 4.0; *Z* = 2.2; *p* = 0.03, one-tailed testing Hedges’ g = 0.68). Exclusion of novices did not lead to other results in this case.

## Discussion

Endoscopic methods and techniques are widely used today and are employed for a variety of procedures worldwide. However, the obvious advantages of these minimally-invasive methods can only be fully exploited if the surgeon is able to cope with the challenges of hand-eye coordination inherent to these methods. This includes an optimized configuration of all relevant components, such as the endoscope placement, the surgeons’ position, and monitor position in relation to each other. In this study, we addressed the question of how to reduce the visual mislocation effect. Typically, the endoscope image is displayed on a monitor at the operating table, causing the surgeon to lose sight of his hands at the operative field. However, the use of a optical see-through head-mounted display (OST-HMD) allows a virtual monitor with the endoscope image to be displayed at any position, while at the same time allowing the surgeon to have visual access to the real environment. This approach allows to eliminate the mislocation effect, as the endoscope image can be projected directly onto the operative field. In addition, OST-HMD devices offer a 3D view of the endoscopic image, which substantially improves spatial orientation and thus hand-eye coordination.

This novel approach was validated in a clinical context with surgeons in a user study. In this study, four positions of a virtual monitor displayed in a OST-HMD were compared. Ten surgeons with varying levels of experience were asked to perform a laparoscopic threading task as quickly as possible, but also with as little interaction force as possible. Our assumption was that compared to +/$-\,60^{\circ }$ positions of the endoscopic picture a front-parallel positioning ($0^{\circ }$) and, in particular, a projection onto the torso, should lead to an improvement in task performance and subjective workload. These assumptions were at least partially confirmed. The times required were actually halved with torso projection compared to the ± 60$^{\circ }$ conditions, but only for the most complex task (Eyelet 3), which placed the greatest demands on bimanual and spatial coordination. This eyelet was rotated by 45$^{\circ }$ to the left from the endoscope’s line of sight. By inserting the needle from the rear, it was particularly challenging for the surgeons to position and orient the needle ideally in the needle holder, which was guided with the right hand. Regarding the interaction forces between needle and eyelets, no effects of the perspectives were found. Further exploratory analyses also showed no effects when, in addition to the reported RMSE metric, other common force metrics to measure surgical fine motor control (mean, peak force, SD of forces; e.g., [14, 15]) were calculated for both the residual force vector as well as for the individual Cartesian axes (x, y, z). Seemingly, the surgeons prioritized contact force minimization over execution duration, leading to significant effects for the latter criterion only. Finally, the more experienced surgeons (> 50 operations) reported that their mental workload was significantly lower with 0$^{\circ }$ and torso positioning than with ± 60$^{\circ }$ positioning. The workload ratings for the novices were on similarly high level in all perspective conditions. The lack of laparoscopic experience seemingly had the effect that the general motor demands of executing the threading task mainly contributed to the subjective workload.

Overall, the technical solution for freely positioning the endoscopic image via OST-HMD was rated positively by the surgeons, particularly the ability to project the image directly onto the torso. This enables almost optimal restoration of hand-eye coordination, as it feels like you are looking directly onto the surgical field. The surgeons mentioned that although torso projection is ideal for hand-eye coordination in demanding tasks, looking down at the torso with a head flexion of >45$^{\circ }$ could be ergonomically unfavorable during lengthy procedures. The presented OST-HMD approach, however, offers an elegant solution to such ergonomic trade-offs, as it allows multiple virtual monitors (e.g., on the torso and at eye level) to be used simultaneously, which can be easily adjusted to procedural demands or individual preferences [16]. Finally, it was noted that the quality of the HMD display was lower compared to that of a standard endoscope monitor.

Although the key findings of the study are based on large effect sizes, the generalizability of the results is clearly limited due to the small and heterogeneous sample. In future studies, a larger sample size, a resolution-optimized HMD, and the option of multiple virtual screens should be investigated.

## Conclusion

Altogether, the presented study provides initial evidence, that virtual monitors based on OST-HMD have the potential to improve ergonomics and performance in conventional laparoscopic procedures, provided that the devices are approved for clinical use and improved in terms of their overall ergonomics and image quality. In addition to the main surgeon, the assistant surgeon could also benefit from such technology in supporting tasks, such as retracting tissue or suction and irrigation. Particularly in robot-assisted laparoscopic surgery (RALS), the bedside assistant takes on many of these tasks, as the main surgeon works remotely from the non-sterile surgeon’s console [17]. Considering the footprint of robotically assisted surgical systems (RASS) as the *da Vinci robotic surgery system*^10^ or the *Hugo™RAS System*,^11^ flexible positioning of the physical monitor to minimize the visual mislocation effect for the bedside assistant would not be possible for any operation room. For this reason, OST-HMDs can play an important role for bedside assistants in RALS. Furthermore, novel system architectures are emerging in RALS. These allow the main surgeon either to switch intraoperatively between conventional laparoscopy at the bedside and robotically assisted laparoscopy from the surgeon’s console (e.g., DEXTER^12^) or to perform the procedure in a solo-surgery manner robotically assisted completely from the bedside (e.g., Maestro Platform™^13^). For these type of RASS architectures, the positioning of the monitors will not only affect the assistant, but as in conventional laparoscopy also the main surgeon, while performing surgical tasks from the bedside.

## Data Availability

Data and codes are available from the authors upon reasonable request.

## Footnotes

1 Insufflated Abdomen with Ports (#2213), The Chamberlain Group, Great Barrington, USA.

2 ATI Industrial Automation, North Carolina, USA.

3 KARL STORZ SE & Co. KG, Tuttlingen, Germany

4 Medtronic plc, Dublin, Ireland.

5 AKTORmed GmbH, Neutraubling, Germany.

6 Microsoft Corporation, Redmond, WA, USA.

7 Epiphan Systems Inc.,Otawa, Canada.

8 https://learn.microsoft.com/en-us/windows/mixed-reality/develop/native/holographic-remoting-overview.

9 Meltytech, LLC, USA.

10 Intuitive Surgical (Sunnyvale, CA, USA).

11 Medtronic plc (Dublin, Ireland).

12 Distalmotion SA (Epalinges, Switzerland).

13 Moon Surgical (Paris, France).
